# Achieving COVID‐19 herd immunity in Ethiopia

**DOI:** 10.1002/puh2.99

**Published:** 2023-06-19

**Authors:** Bezawit Kassahun Bekele, Abdulbasit Opeyemi Muili, Iyaketing Akpan Udom, Lizeth Hernández‐Rubio, Yidnekachew Girma Mogessie, Shewit Yehuala Wubneh, Goodness Ogeyi Odey, Dawit Tesfagiorgis Mengesha

**Affiliations:** ^1^ College of Health Sciences Addis Ababa University Addis Ababa Ethiopia; ^2^ Department of Medicine Ladoke Akintola University of Technology Ogbomoso Nigeria; ^3^ Department of Physical and Health Education University of Uyo Uyo Nigeria; ^4^ Johns Hopkins Bloomberg School of Public Health Johns Hopkins University Baltimore Maryland USA; ^5^ Johns Hopkins Carey Business School Johns Hopkins University Baltimore Maryland USA; ^6^ St. Paul's Hospital Millennium Medical College Addis Ababa Ethiopia; ^7^ Refugees and Returnees Service Addis Ababa Ethiopia; ^8^ Department of Public Health, College of Medical Sciences University of Calabar Calabar Nigeria

**Keywords:** COVID-19, Ethiopia, herd immunity, population health, vaccine efficacy, vaccine hesitancy, pandemic, Africa

## Abstract

COVID‐19 has greatly impacted the society and economy of Ethiopia, with a total of 499,493 cases confirmed and a death toll of 7572 as of February 2023. The country began COVID‐19 vaccination in March 2021 and delivered about 52 million doses as of July 2022. The government liaised with the COVAX (COVID‐19 Vaccines Global Access) facility, the African Vaccine Acquisition Trust, and other donors to acquire the vaccine doses. However, in July 2022, Ethiopia achieved only 45 persons per 100 population coverage when the global average of doses administered was 167 per 100 population. These figures grossly fall behind the requirements for achieving herd immunity. The major challenges noted are vaccine hesitancy ranging from 14.1% to 68.7%, including that among healthcare workers (HCW). There was a lack of physical infrastructure and personnel to distribute the vaccines and to administer the doses to populations who had challenges accessing healthcare services. There was heavy disinformation regarding COVID‐19 vaccines. The various government ministries including the Ministry of Health (one government approach) and the regional and zonal health offices should coordinate for efficient and equitable vaccine distribution and build community trust by working with village leaders, religious groups, and media outlets. There is a need to continue efforts on providing an awareness of the importance of COVID‐19 vaccination and herd immunity. We review and dissect the COVID‐19 vaccination efforts in Ethiopia and provide recommendations for tackling these challenges, including equity in the distribution of vaccines and proper awareness of the disease to achieve herd immunity.

## INTRODUCTION

Ethiopia is home to around 96 million people, of which nearly 13 million are children under 5 years old [1]. It is one of the African countries facing the double burden of communicable and noncommunicable diseases. The COVID‐19 pandemic has tremendously impacted people's livelihood, health, and development. The pandemic has exacerbated the existing food insecurity and undermined the livelihood of the people in Ethiopia. The pandemic also had a significant impact on Ethiopia's healthcare service delivery as pandemic measures have taken precedence over routine healthcare services, such as maternal and child care, and immunization. Hospitals were also struggling to meet the increased demand for providing services, and health workers were overburdened due to increased workloads in the background of inadequate supply of personal protective equipment (PPE) [2].

The first case of COVID‐19 was detected on March 13, 2020, and as of February 3, 2023, a total of 499,493 cases were confirmed, and 7572 deaths were reported in Ethiopia [3]. Throughout the pandemic, screening protocols, public awareness, and health education have been key to containing the spread of the virus. Aside from public awareness and education programs, vaccination is the most successful, safe, and effective method of containing the COVID‐19 pandemic [4]. The countrywide immunization effort in Ethiopia began on March 13, 2021, targeting high‐risk groups (i.e., elderly, patients with chronic diseases, and healthcare workers [HCWs]). The immunization campaign was expanded to include everyone aged 12 or older on November 16, 2021. We review the COVID‐19 vaccination efforts in Ethiopia and identify the gaps and challenges in achieving herd immunity in the country.

## COVID‐19 VACCINATION EFFORTS

Following the discovery of the COVID‐19 vaccine, numerous efforts are being implemented to deliver the vaccines to the farthest regions in underprivileged communities around the world. In 2021, World Health Organization (WHO) collaborated with relevant organizations, including COVAX (COVID‐19 Vaccines Global Access), the African Vaccine Acquisition Trust (AVAT), and many other stakeholders to provide vaccinations to low‐ and‐middle‐income countries like Ethiopia [5]. Early in March 2021, through the COVAX facility, Ethiopia received the first large shipment of 2.2 million doses of the AstraZeneca vaccine, marking the beginning of the country's COVID‐19 vaccination campaign [6]. Although more than 3.84 billion (about 50% of the world's population) individuals worldwide had received one dosage of the COVID‐19 vaccine by October 26, 2021, the vaccination coverage was less than 3% among 24 African countries and only 0.9% in Ethiopia [7].

To address the low vaccination rate in Ethiopia, the Federal Ministry of Health (MoH) in November 2021 deployed over 28,000 vaccinators and more than 6.2 million doses of COVID‐19 vaccines, namely, Sinopharm, AstraZeneca, Johnson & Johnson/Janssen, and Pfizer‐BioNTech. Upon the recommendations from the National Immunization Technical Advisory Group, heterologous prime‐boost vaccination, which means using different types of vaccines for the first and second doses, was implemented to optimize vaccine efficacy and overcome supply shortages. Ethiopia received assistance from health and immunization partners like UNICEF (United Nations Children's Fund), AMREF (African Medical and Research Foundation), Resolve to Save Lives Fund, Save the Children, and others working in partnership with the government and manufacturers to ensure equal access to the COVID‐19 vaccine [8]. As of July 30, 2022, a total of 52,509,414 vaccine doses were administered in Ethiopia with a coverage of 45 per 100 eligible population. This figure was much lower compared to the total vaccine doses of 167 per 100 population globally and 49 per 100 population in Africa in the same time frame [3]. More efforts, such as the expansion of vaccination sites, the effective use of available stocks, community mobilization, and addressing misinformation, were major barriers to higher vaccine uptake in Ethiopia.

## NATIONAL HERD IMMUNITY GOALS

Achieving herd immunity against COVID‐19 may be a challenge due to the virus's ability to mutate and the lack of long‐lasting protection from previous infections [9]. The WHO set out policies to help improve herd immunity globally. The major goal was to increase vaccination coverage, minimize deaths, and stop new emerging variants by mid‐2022 [10]. For COVID‐19, it is estimated that 94% of the population needs to be immune to stop the spread of the virus. Herd immunity can be acquired with a coverage of an adequate proportion of the population with vaccines [11].

One of the main factors that hinder the achievement of herd immunity in Ethiopia is vaccine hesitancy reported to be at 14.1%–68.7% of the population [12]. The primary reasons include a lack of awareness, doubts about the vaccine's effectiveness, and negative attitudes toward the vaccine [13]. A study among HCWs in Addis Ababa, Ethiopia found that nearly two thirds reported COVID‐19 vaccine hesitancy. Lack of trust in the vaccine's benefits, lack of trust in the government and science to produce safe and effective vaccines, and concern about vaccine safety were some of the factors among Ethiopian HCWs [14].

Compared to high‐income countries, low‐ and middle‐income countries like Ethiopia face the challenge of equitable access to COVID‐19 vaccines. The lack of technology transfer and support for local vaccine manufacturers, the capacity to implement effective vaccination programs, and the limited resources and capacity to buy and distribute vaccines are major barriers to achieving higher vaccination coverage (Table 1). Ethiopia is a large country with an uneven population distribution, making it difficult to reach remote areas and deliver vaccines over long distances. This is exacerbated by a shortage of personnel needed to distribute and administer the vaccines [15].

**TABLE 1:** Challenges and strengths in achieving COVID‐19 herd immunity in Ethiopia [13, 15–17].

Strengths	Challenges
Provision of essential medical supplies, such as personal protective equipment, ventilators, and other materials by the Ethiopian government Enforcement of strict containment measures, including the closure of schools and workplaces and banning social activities Implementation of extensive testing and contact tracing system and nationwide campaign to raise awareness about the virus Approval of a US $500 million budget by the Ethiopian Prime Minister to support the economy and vulnerable populations during the pandemic	Vaccine hesitancy due to a lack of awareness, doubts about the vaccine's effectiveness, and negative attitudes toward the vaccine Inequitable access to the COVID‐19 vaccines, limited capacity to purchase vaccines, the lack of technology transfer and support for local vaccine manufacturers, and the lack of capacity to implement vaccination programs Logistical barriers, the lack of physical resources, and personnel trained in administering vaccines

## RECOMMENDATIONS

To tackle the COVID‐19 vaccine hesitancy in Ethiopia, the Ministry of Health along with the public health institutes, and regional and zonal health offices should work on providing accurate and timely information about the safety of the vaccine, including its risks and benefits. The task force should also work with community leaders, religious groups, health workers, and media outlets to promote positive attitudes and behaviors toward COVID‐19 vaccination. To successfully deliver vaccines across the country, both logistical support in the form of physical resources and personnel trained in providing vaccinations are required. The COVID‐19 vaccination, policymakers, and vaccine‐campaign program planners should also ensure equity by making vaccines accessible to marginalized populations.

## CONCLUSION

COVID‐19 had major impacts on the socioeconomic and health infrastructure in Ethiopia. The country could vaccinate only 45 per 100 people due to vaccine hesitancy and unequal vaccine distribution. To achieve COVID‐19 herd immunity, there is a need to enhance equitable distribution and achieve greater vaccine coverage with a special focus on the difficult‐to‐reach and marginalized population. There is a need to continue the awareness of the importance of herd immunity through news outlets, televisions, and radios to tackle the increasing volumes of fake news.

## AUTHOR CONTRIBUTIONS

*Conceptualization; project administration; supervision; writing—original draft; writing—review and editing*: Bezawit Kassahun Bekele. *Writing—original draft; writing—review and editing*: Abdulbasit Opeyemi Muili and Iyaketing Akpan Udom. *Writing—review and editing*: Lizeth Hernández‐Rubio, Yidnekachew Girma Mogessie, Shewit Yehuala Wubneh, and Dawit Tesfagiorgis Mengesha. *Conceptualization; writing—review and editing*: Goodness Ogeyi Odey.

## CONFLICT OF INTEREST STATEMENT

Goodness Odey is an Editorial Board member of Public Health Challenges and coauthor of this article. She was excluded from editorial decision‐making related to the acceptance of this article for publication in the journal.

## FUNDING INFORMATION

There is no funding for the development of this paper.

## Data Availability

No database or primary data was used in preparing the manuscript.
